# Modulating the RFamide-related peptide-3/G protein-coupled receptor 147 signaling pathway with nourishing Yin-removing fire herbal mixture to alleviate precocious puberty in female rats: An experimental study

**DOI:** 10.18502/ijrm.v22i1.15240

**Published:** 2024-02-23

**Authors:** Xiaoli Dai, Yuanyuan He, Suhuan Li, Yanyan Sun, Wen Sun, Zhanzhuang Tian, Ph.D., Jian Yu, Ph.D., Nurgul Ablakimova, Yonghong Wang

**Affiliations:** ^1^Traditional Chinese Medicine Department, Children's Hospital of Fudan University, Shanghai, China.; ^2^Yantaishan Hospital, Shangdong, China.; ^3^Department of Integrative Medicine and Neurobiology, Fudan University, Shanghai, China.; ^4^Department of Pharmacology, West Kazakhstan Marat Ospanov Medical University, Aktobe, Kazakhstan.

**Keywords:** Nourishing Yin-removing fire, RFamide-related peptide-3, G protein-coupled receptor 147, Hypothalamus, Puberty, Precocious.

## Abstract

**Background:**

Precocious puberty (PP) involves early activation of the hypothalamic gonadotropin-releasing hormone (GnRH) generator. The RFamide-related peptide/G protein-coupled receptor 147 (*RFRP3/GPR147*) signaling pathway is vital in inhibiting GnRH and delaying puberty onset. The nourishing Yin-removing fire (NYRF) herbal mixture has shown promising results in treating PP.

**Objective:**

This study aimed to assess the impact of the NYRF herbal mixture on the *RFRP3/GPR147* signaling pathway in the hypothalamus and its potential in alleviating PP in female rats.

**Materials and Methods:**

In a controlled experiment, 24 female Sprague-Dawley rats (11.20 ± 0.69 gr, postnatal day [PD5]) were divided into normal, model, normal saline, and NYRF groups (n = 6/each). PP was induced in the model, normal saline, and NYRF groups by subcutaneous injection of danazol at PD5. The NYRF herbal mixture or normal saline was administered from PD15. Serum sex hormone levels and hypothalamic samples were collected for mRNA and protein expression at PD30.

**Results:**

In the model group, hypothalamic GnRH and kisspeptin levels increased, while RFRP3 and GPR147 levels decreased, luteinizing hormone levels elevated, reproductive organ coefficients increased, and the vagina opened earlier compared to the normal group. Conversely, the NYRF group exhibited lower GnRH and kisspeptin levels but higher RFRP3 levels in the hypothalamus. Serum luteinizing hormone levels were reduced, reproductive organ coefficients were reduced, and the vaginal opening was delayed compared to the model and normal saline groups.

**Conclusion:**

The NYRF herbal mixture delayed sexual development in rats with PP by hypothalamic upregulating RFRP3 and downregulating GnRH and kisspeptin.

## 1. Introduction

Precocious puberty (PP) is a prevalent endocrine disorder in children with premature onset of secondary sexual characteristics (1). In China, the study estimated a prevalence of 6.19% (11.47% girls, 3.26% boys) based on a survey of 4058 primary school students (2). The onset of puberty is a complex biological process under the control of neuroendocrine pathways with multiple factors involved in regulating the hypothalamic-pituitary-gonadal (HPG) axis (3). Puberty initiation relies on crucial mechanisms such as activating the gonadotropin-releasing hormone (GnRH) pulse generator and the emergence of GnRH peaks, which is the key player in puberty onset, meanwhile, the disruption of GnRH release leads to gonadotropin-dependent PP (4).

Kisspeptin, a neuropeptide encoded by the *Kiss1* gene, plays a vital role in activating the hypothalamic-HPG axis. By stimulating the hypothalamus, *Kiss1* acts upstream of GnRH and is recognized as a critical regulator of GnRH secretion, which may help control the timing of puberty (5). GnRH neuron regulation involves excitatory and inhibitory networks, with gonadotropin-inhibitory hormone (GnIH) playing a significant inhibitory role (6). As an inhibitor of HPG axis, GnIH was the first to be identified in the Japanese quail, marking its initial discovery (7). GnIH could directly inhibit GnRH release in the quail brain, which could open a new window in the knowledge of HPG axis function (8). The RFamide-related peptide (RFRP) precursor, the mammalian equivalent of GnIH, produces 2 neuropeptides: RFRP1 and RFRP3 (9).

The G protein-coupled receptor 147(GPR147) receptor, a novel GPR, has been identified in the Japanese quail. GPR147binds to RFRP3 on some GnRH and kisspeptin neurons. Additionally, RFRP3 may influence GnRH and kisspeptin neurons (10). Meanwhile, they conducted intracerebroventricular injections of RFRP3 in female rats, observing downregulation of GnRH and *Kiss1* mRNA expression in the hypothalamus and significant delays in puberty onset (11). These findings suggest that RFRP3/GPRl47 signal pathway can function as an inhibitory regulator of reproductive capability, by regulating gonadotrophin synthesis and release through its effects on the HPG axis.

The nourishing Yin-removing fire (NYRF) herbal mixture (Shanghai pharmacists system number Z05170908, consists of *Rehmannia glutinosa* [Sheng-Di-Huang], *Scrophularia buergeriana* [Xuan-Shen], and *Anemarrhena asphodeloides* [Zhi-Mu], etc.), an experienced prescription originating from the famous TCM experts, has been used to treat PP for decades (12). We have observed that it could mitigate the progression of sexual characteristics in PP girls with kidney-yin deficiency and fire hyperactivity (13). In previous experimental studies, we have found that NYRF mixture could suppress the expression of kisspeptin and GnRH in the hypothalamus of female PP rats (14).

Considering the multi-target therapeutic effects of the Chinese herbal mixtures, we investigated the modulation of NYRF herbal mixture to the RFRP3/GPR147 signal pathway in the hypothalamus of PP female rats, in order to provide additional insights into the therapeutic mechanism of the NYRF herbal mixture in the context of PP.

## 2. Materials and Methods

### Animal procurement and housing

Newborn Sprague-Dawley rats, along with their mother, were procured from the Laboratory Animal Business Department, of Shanghai Institute of Family Planning (license number SCXK [Shanghai] 2018–0006) to conduct an experimental study. The rats were housed under standard conditions at the Department of Integrative Medicine and Neurobiology, Fudan University, Shanghai, China which included a temperature of 22 ± 2 C, humidity of 30–40%, and a 12-hr light/dark cycle. Adequate food and water were provided to the rats ad libitum.

### Preparation of the herbal mixture

The initial formulation of the NYRF herbal mixture, formulated by the Department of Integrative Medicine at Children's hospital of Fudan University (Shanghai Medicine: Z05170908 consisted of ingredients such as *Rehmannia glutinosa*, Scrophulariae Radix [dried root of *Scrophularia ningpoensis*], and Phellodendri Amurensis Cortex [dried bark of *Phellodendron chinense*], etc.) (Table I). The reparation method employed in this study follows the protocol outlined in our prior research (15).

To obtain the NYRF herbal mixture extract, the traditional method of water extraction followed by alcohol precipitation was chosen (16). The crude drugs were decocted using a thermostat electric set (Zhengzhou Great Wall Scientific Industrial & Trade Co. Ltd., Zhengzhou, China) at 100 C for 40 min. The set was refilled with water, and the decoction continued for another 40 min. The extractive liquid was then accumulated and concentrated on a rotary evaporator (Buchi, Switzerland) for 15 min at 4 C at 1000 rpm/min. Absolute ethanol was slowly added to the extractive liquid until the ethanol concentration reached 60%. The mixture was then incubated at 4 C for 72 hr to remove the impurities that might dissolve in ethanol and precipitate to the bottom. The supernatant was extracted and the ethanol was subsequently removed from it using a rotary evaporator for 15 min at 4 C at 1000 rpm/min. Following this method, the final drug was obtained at a concentration of 2.7 gr per/ml (15).

### Study design, animal grouping, and drug administration

In an experimental study, 24 rats were allocated randomly into 4 groups (n = 6): the normal group, the model group, the normal saline group, and the NYRF group. Except for the rats in the normal group, all the other groups were administered a single subcutaneous injection of 300 µg/25 µL danazol (dissolved in ethylene glycol: ethanol, 1:1) on postnatal day (PD)5 to induce the PP model (17). From PD15 to PD30, the rats in the NYRF group received an oral administration of the NYRF extract twice daily (1 mL/100 gr), while the rats in the normal saline group were given an equal volume of saline. No additional criteria were employed for participant inclusion or exclusion.

To minimize potential confounders, each participant's order of treatments and measurements were randomized. Animal cages were randomly assigned and periodically rotated to mitigate any potential bias associated with cage location. These measures were taken to minimize extraneous factors and enhance the study's internal validity. At the stage of group allocation, the researchers responsible for assigning the rats to their respective groups were aware of the group assignments. However, during the experiment, the outcome assessment, the subsequent data analysis, and the individuals involved were blinded to the group allocation to minimize potential biases and ensure objective evaluation and analysis of the results.

### Sex character observation, samples management

Daily monitoring of vaginal opening commenced from PD15, and the timing of vaginal opening was documented. After the day of vaginal opening, vaginal smears were taken regularly every morning to observe estrous cycle phases until PD30. There are 4 phases in the entire estrous cycle: proestrus, estrus, metestrus, and diestrus, which last 4–5 days. The vaginal secretions comprise 3 types of cells: leucocytes, cornified epithelial cells, and nucleated epithelial cells (18). During the estrus phase, there is a high presence of enucleated cornified epithelial cells. The cytoplasm is granular, and the cells have an irregular shape. At PD30, all rats (n = 6 in each group) were anesthetized through intraperitoneal injection of pentobarbital sodium (50 mg/kg) (19). Serum samples were collected and stored at -80 C for subsequent hormone level analysis. The hypothalamus was also collected and stored at -80 C, whereas the uterus and ovaries were excised and weighed.

### Organ coefficients, hormone level detection

The uterine and ovarian coefficients were computed (n = 6 in each group) utilizing the organ index formula: ([organ wet weight (g)/body weight (g)] × 10^4^). To determine the levels of estradiol (E2), luteinizing hormone (LH), and follicle-stimulating hormone (FSH) in the serum samples, enzyme-linked immunosorbent assay kits (eBioscience, Affymetrix, USA) were employed.

### Quantitative real-time polymerase chain reaction (qRT-PCR)

Total RNA was extracted using the Trizol method and the concentration and purity were determined using the ultraviolet spectrophotometer (OD ${\;}_{260}$ /OD ${\;}_{280}$ , 1.8 ∼ 2.0). Then, the Real-Time PCR Master Mix Code (Toyobo, Japan) was used to reverse transcribe the RNA into cDNA. The primer sequences, as listed in table II, were designed and synthesized by Shanghai Jierui. The qRT-PCR system was conducted following the ReverTra Ace qRT-PCR kit protocol (Toyobo, Japan), which involved an initial step at 95 C for 180 sec, followed by 40 cycles of 95 C for 5 sec and 60 C for 30 sec. The relative expression of mRNA in the hypothalamus of rats (n = 6/each) was determined using the 2 ${\;}^{-\;\;Ct}$ method.

### Protein analysis by western blotting

Protein extraction from the hypothalamus of rats (n = 6/each) was conducted using radioimmunoprecipitation assay (RIPA) buffer, and the protein concentration was determined using a bicinchoninic acid kit (Biosharp, Hefei, China). Gels were prepared and concentrated accordingly to separate the proteins based on their molecular weight. The protein samples were then subjected to electrophoresis and transferred onto polyvinylidene fluoride membranes. These membranes were blocked with 5% bovine serum albumin (BSA) in tris-buffered saline containing 0.05% tween 20 (TBST) and subsequently incubated overnight at 4 C with primary antibodies, including anti-kisspeptin antibody (Abcam, US), anti-RFRP3 antibody (Santa Cruz, US), and anti-GPR147 antibody (Biorbyt, UK). After 3 washes with TBST for 10 min each, the membranes were exposed to suitable secondary antibodies for 1 hr. Chemiluminescence detection was performed using an electrochemiluminescence reagent, and the intensity of the protein bands was quantified using ImageJ software.

**Table 1:** Ingredients for the nourishing Yin-removing fire mixture used to treat PP rats, per 1000 ml of water


**Chinese name**	**Common name**	**Family**	**Scientific name**	**Weight (g)**
**Mai ya**	Barely	Gramineae	*Hordeum vulgare L.*	30
**Gui jia**	Plastron of fresh-water tortoise	Testudinidae	*Chinemys reevesii* (Carapax et Plastrum Testudinis)	12
**Sheng Di huang**	Rehmannia root	Scrophulariaceae	*Rehmannia glutinosa*	15
**Xuan shen**	Buerger's Figwort	Scrophulariaceae	*Scrophularia buergeriana*	9
**Zhi mu**	Zhimu	Liliaceae	*Anemarrhena asphodeloides*	9
**Huang bai**	Phellodendron bark	Rutaceae	*Cortex phellodendri*	9
**Dan pi**	Moutan	Ranunculaceae	*Paeonia suffruticosa Andr*	9
**Ze xie**	Alisma oriental	Alismataceae	*Alisma plantago-aquatica L. var. orientale Sam*	9
**Xia ku cao**	Common self-healing	Lamiaceae	*Prunella vulgaris L.*	9

**Table 2:** Primers used to evaluate the effect of nourishing Yin-removing fire herbal mixture that alleviates PP in female rats by modulating RFamide-related peptide-3/G protein-coupled receptor 147 signaling pathway in the hypothalamus


	**Sequence (5 ' → 3 ' )**	
**Gene**	**Forward**	**Reverse**
	*GAPDH*	ACTTTGGCATCGTGGAAGGG	TGCAGGGATGATGTTCTGGG
*GnRH*	CACTGGTCCTATGGGTTGCG	TCCCTAAGAGGTGAACGGGG
*RFRP3*	CCTGAGGTTTGGGAGGAACATA	GTGTCTTGGTGATGCGTCTG
*Kiss1*	GGTATGCAGAGAGCAAGCCT	GATCAGGCGACTGCGGG
*GPR147*	CCCGAGAGGAGCATCACTTC	GTAGATGTGCGCGAAGAGCA
GAPDH: Glyceraldehyde-3-phosphate dehydrogenase, GnRH: Gonadotropin-releasing hormone, RFRP3: RFamide-related peptide-3, Kiss: Kisspeptin, GPR147: G protein-coupled receptor 147		

### Ethical considerations

The study was approved by the Ethical Committee of the Children's hospital of Fudan University, Shanghai, China (No: [2020] 211). The animals involved in the study were treated and handled in accordance with the Ethical Guidelines of Fudan University, Shanghai, China.

### Statistical analysis

The study outcomes encompassed several parameters, including the timing of vaginal opening, ovarian coefficient, uterine coefficient, LH, FSH, LH/FSH ratio, E2, *GnRH* mRNA expression, *Kiss1* mRNA expression, kisspeptin protein expression, *RFRP3* mRNA expression, RFRP3 protein expression, *GPR147* mRNA expression, and GPR147 protein expression. The comparisons between the groups were conducted using one-way ANOVA and post hoc Tukey test. Pearson correlation analysis was utilized to evaluate correlations. A significance level of p < 0.05 was considered statistically significant. The calculation was based on the protein expression data obtained after the study. Statistical analyses were carried out using SPSS for Windows program, version 22 (SPSS Inc., Chicago, Illinois, USA). Group means and standard errors were reported in both text and graphs using GraphPad Prism version 9.0 for Windows (GraphPad Software Inc., San Diego, CA, USA).

The sample size calculation for this study was conducted using G*Power 3.9.11.2 software, considering a desired statistical power of 0.8, an alpha error level of 0.05, and employing a one-way ANOVA test for 4 groups.

## 3. Results

### Assessment of vaginal opening time, sex organ development, estrous cycle phases, body weight in different groups

The timing of vaginal opening was carefully documented throughout the experimental period. No instances of vaginal opening were observed in the normal group until PD30. In contrast, the model group exhibited significantly early vaginal opening compared to the normal group. Notably, the NYRF group showed a considerably delayed time of vaginal opening in comparison to both the model group (p = 0.01, Table III) and the normal saline group (p = 0.01).

The organ coefficients of the uterus and ovaries were analyzed to evaluate reproductive organ development. Remarkably, the ovarian and uterine coefficient of rats in the model group exhibited a notable increase compared to those in the normal group (p = 0.004; p = 0.000). In contrast, the NYRF group displayed lower ovarian and uterine coefficients than the model group (p = 0.008; p = 0.003) and the normal saline group (p = 0.026; p = 0.001).

After the day of vaginal opening, the daily vaginal smears were examined to observe estrous cycle phases until PD30. The time of the first estrus phase in the model group and the normal saline group was (29.67 ± 0.21) days and (29.50 ± 0.22) days, respectively. No statistically significant differences were observed in the 2 groups (p = 0.599). However, until PD30 the first regular estrous cycle was still not observed in 4 groups. Considering the potential influence of body fat on *Kiss1* expression, the weights of the animals in the 4 experimental groups were recorded at PD5 and PD30, when the samples were obtained. No statistically significant differences were observed in different rat groups at PD5 and PD30 (p = 0.993 and p = 0.928, respectively) (Table IV).

### Analysis of hormone levels in rats from various groups

Compared to the normal group, rats in the model group displayed elevated levels of LH and LH/FSH, while exhibiting reduced levels of FSH (all p = 0.000; Table V). Notably, rats in the NYRF group exhibited lower levels of LH and LH/FSH than the model and normal saline groups (all p = 0.000). Interestingly, no significant differences were observed in E2 levels between the groups (p = 0.975, the normal group vs. the model group; p = 0.939, the NYRF group vs. the model group; p = 0.960, the NYRF group vs. the normal saline group).

### Analysis of GnRH and kisspeptin expression in the hypothalamus of rats from different groups

**Figure 1:**
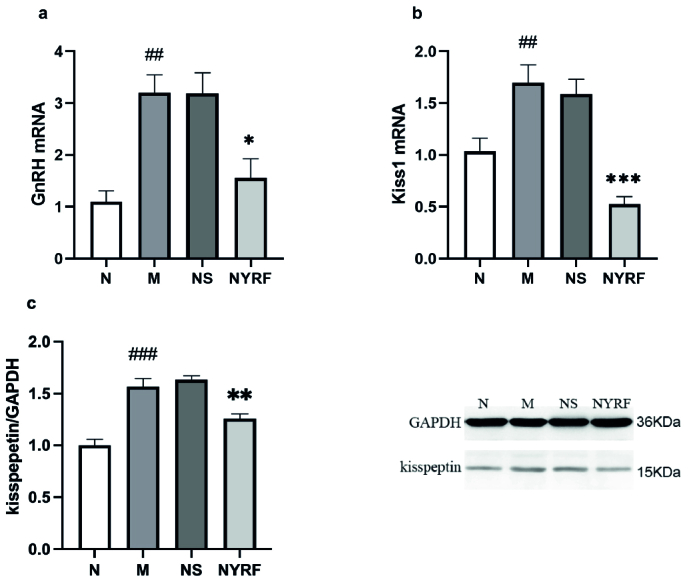
Analysis of gonadotropin-releasing hormone (GnRH*)* and kisspeptin expression. a) *GnRH* mRNA expression. b) *Kiss1* mRNA expression. c) kisspeptin protein expression. Western blot gels of kisspeptin are also presented. Data are Means ± SEM. ${\;}^{\#\#}$ P < 0.01, ${\;}^{\#\#\#}$ P < 0.001 the model group (M) vs. the normal group (N). *P < 0.05, **P < 0.01, ***P < 0.001 the normal saline group (NS) vs. the nourishing Yin-removing fire group (NYRF).

Compared to the normal group, the model group exhibited a significant increase in the expression of *GnRH* and *Kiss1* mRNA in the hypothalamus (p = 0.001 and p = 0.012, respectively; Figure 1). The expression of *GnRH* mRNA in the NYRF group was lower than that in the model group, and the normal saline group (p = 0.013 and p = 0.014, respectively), and a similar trend was observed for *Kiss1* mRNA expression (p = 0.000 and p = 0.000, respectively). To further investigate the kisspeptin expression, we analyzed the protein levels using Western blot analysis. The protein expression of kisspeptin showed a consistent pattern with the mRNA results. The model group displayed higher levels of kisspeptin protein than the normal group (p = 0.000). In contrast, the NYRF group exhibited lower levels of kisspeptin protein compared to both the model group (p = 0.005) and the normal saline group (p = 0.001).

### Analysis of *RFRP3* and *GPR147* expression in the hypothalamus of rats from different groups

**Figure 2:**
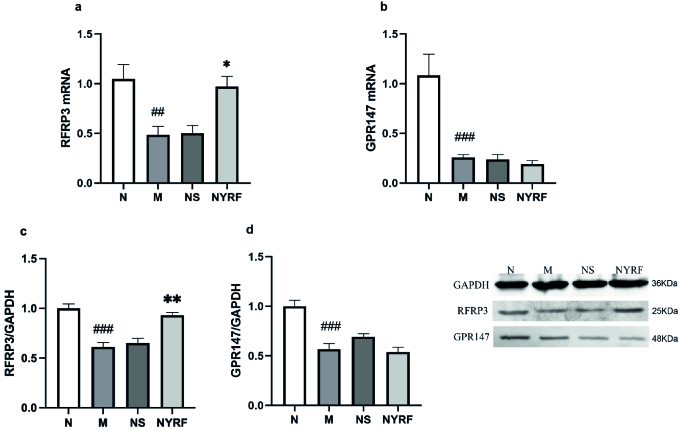
Analysis of *RFamide-related peptide-3* (*RFRP3*) and *G protein-coupled receptor 147* (*GPR147*) expression. a) *RFRP3* mRNA expression. b) RFRP3 protein expression. c) *GPR147* mRNA expression. d) GPR147 protein expression. Western blot gels are also presented. Data were presented as Means ± SEM. ${\;}^{\#\#}$ P < 0.01, ${\;}^{\#\#\#}$ P < 0.001 the normal group (N) vs. the model group (M). *P < 0.05, **P < 0.01 the normal saline group (NS) vs. the nourishing Yin-removing fire (NYRF) group.

As per our expectations, compared to the normal group, rats in the model group exhibited a significant decrease in both *RFRP3 *mRNA and protein levels at PD30 (p = 0.006 and p = 0.000, respectively; Figure 2). However, after treatment with the NYRF herbal mixture, the mRNA levels of *RFRP3* increased in the NYRF group compared to the model and normal saline groups (p = 0.19 and p = 0.023, respectively), as did the protein levels of RFRP3 in the NYRF group compared to the model and normal saline groups (p = 0.000 and p = 0.645, respectively). Furthermore, we observed considerably lower levels of *GPR147* mRNA and protein expression in the model group compared to the normal group (p = 0.000 for both). However, no significant differences were found between the NYRF group, the model group, and the normal saline group in terms of *GPR147* mRNA expression (p = 0.974, the NYRF group vs. the model group; p = 0.991, the NYRF group vs. the normal saline group) and protein expression (p = 0.977, the NYRF group vs. the model group; p = 0.171, the NYRF group vs. the normal saline group).

### Correlation analysis of *RFRP3* with *GnRH*, *Kiss1* mRNA, and LH levels

We evaluated the correlation between various measured parameters. Notably, *RFRP3* exhibited a significant inverse correlation with *Kiss1* mRNA (r = -0.55; p = 0.005), *GnRH* mRNA (r = -0.56; p = 0.005), and LH levels (r = -0.63; p = 0.000).

**Table 3:** Comparison of the vaginal opening time and the organ coefficient to evaluate the effect of NYRF herbal mixture that alleviates PP in female rats by modulating RFamide-related peptide-3/G protein-coupled receptor 147 signaling pathway in the hypothalamus


**Group (n = 6)**	**Time of vaginal opening (days)**	**Ovarian coefficient (×10^4^)**	**Uterine coefficient (×10^4^)**
**Normal**	- 2.20 ± 0.13	3.89 ± 0.92
**Model**	22.67 ± 0.49	3.19 ± 016##	16.95 ± 1.44###
**Normal saline**	22.83 ± 0.48	3.05 ± 0.25	17.81 ± 1.74
**Nourishing Yin-removing fire**	25.67 ± 0.56*	2.26 ± 0.16*	9.33 ± 0.99**
Data were presented as Means ± SEM. ${\;}^{\#\#}$ P < 0.01, ${\;}^{\#\#\#}$ P < 0.001 the model group vs. The normal group. *P < 0.05, **P < 0.01 the NYRF group vs. the normal saline group			

**Table 4:** Comparison of body weight at postnatal day 5 and 30 in different groups to evaluate the effect of nourishing Yin-removing fire herbal mixture that alleviates PP female rats by modulating RFamide-related peptide-3/G protein-coupled receptor 147 signaling pathway in the hypothalamus


**Group (n = 6)**	**Body weight at PD5 (g)**	**Body weight at PD30 (g)**
**Normal**	11.15 ± 0.44	107.27 ± 2.54
**Model**	11.27 ± 0.17	106.75 ± 2.02
**Normal saline**	11.17 ± 0.27	105.68 ± 1.86
**Nourishing Yin-removing fire**	11.20 ± 0.26	107.62 ± 2.22
Data were presented as Means ± SEM. No statistically significant differences were observed at PD5 and PD30 (p = 0.999 and p = 0.928, respectively). PD: Postnatal day		

**Table 5:** Comparison of the serum hormone levels to evaluate the effect of nourishing Yin-removing fire herbal mixture alleviates precocious puberty female rats by modulating RFamide-related peptide-3/G protein-coupled receptor 147 signaling pathway in the hypothalamus


**Group (n = 6)**	**LH (ng/L)**	**FSH (IU/L)**	**LH/FSH**	**E2 (ng/L)**
**Normal**	33.39 ± 0.90	26.73 ± 0.63	1.26 ± 0.06	65.95 ± 3.20
**Model**	45.29 ± 1.23###	15.47 ± 0.88###	2.98 ± 0.21###	64.38 ± 2.56
**Normal saline**	41.69 ± 0.69	19.01 ± 0.64	2.21 ± 0.10	64.68 ± 2.68
**Nourishing Yin-removing fire**	34.69 ± 1.30**	26.51 ± 0.80***	1.31 ± 0.16***	66.55 ± 2.23
Data were presented as Means ± SEM. LH: Luteinizing hormone, FSH: Follicle-stimulating hormone, E2: Estradiol. ${\;}^{\#\#\#}$ P < 0.001 the model group vs. the normal group. **P < 0.01, ***P < 0.001, the NYRF group vs. the normal saline group				

## 4. Discussion

These studies showed that puberty onset might occur relative to the downregulated expression of *RFRP3*, and thus we hypothesized that the mechanism of NYRF herbal mixture delays the PP. The mammalian reproductive system is regulated by HPG axis, in which hypothalamic GnRH neurons release pulses of GnRH into the pituitary gland, playing a crucial role in reproduction and puberty (20). Previous studies have usually focused on the upregulatory mechanisms of pubertal initiation such as kisspeptin and GnRH. It has been confirmed that kisspeptin is a neuropeptide that plays a key role in reproductive regulation. Interestingly, kisspeptin, encoded by the *Kiss1* gene, is a positive control for GnRH release. It also promotes the secretion of GnRH (21).

*RFRP3*, also known as *GnIH*, inhibits gonadotropin release and is produced in specific brain regions, exerting an inhibitory effect on gonadal function (22). As one of the inhibitory regulators, it regulates the synthesis and secretion of GnRH. These signaling molecules play important roles in the development of PP (23). The *RFRP* neurons are primarily distributed in the dorsomedial nucleus of the hypothalamus in rats, and their nerve fibers project to GnRH and kisspeptin neurons, which also express *GPR147*. These morphological connections provide evidence for the regulation of GnRH and kisspeptin neurons (24). In the PP model induced by danazol, a decline in* RFRP3* and *GPR147* expression has been observed (25). Similarly, in this study, we found a significant downregulation of *RFRP3* and *GPR147 *expression in the hypothalamus of the model group rats, suggesting that the reduced inhibitory effect of *RFRP3/GPR147* on GnRH and kisspeptin neurons contributed to the promotion of PP female rats. Those studies have highlighted the involvement of the *RFRP3/GPR147* signaling pathway in the regulation of reproduction and PP pathological process. Previous clinical studies have shown that NYRF herbal mixture delays the onset of advanced puberty, increases final height in adulthood, and delays the age of menarche in girls (14). In experimental studies, we also found that NYRF herbal mixture could affect the hypothalamic kisspeptin signaling pathway meanwhile, downregulating the increased GnRH expression for treating PP with the NYRF herbal mixture.

It has not been previously reported whether the mechanism of treatment of PP by the NYRF herbal mixture is through the *RFRP3/GPR147* signaling pathway. In this study, after the treatment with the NYRF herbal mixture, we found that both the mRNA and protein levels of *RFRP3* were increased in the NYRF group compared with the model and normal saline groups at PD30. However, the lower expression levels of kisspeptin and GnRH in the hypothalamus, as well as decreased LH levels, reproductive organ coefficients, and delayed vaginal opening, suggested that the NYRF herbal mixture may delay the onset of puberty in female rats by upregulating hypothalamic *RFRP3* expression.

The induction of PP in female rats through subcutaneous injection of danazol has been well-documented. This experimental model is characterized by early vaginal opening, elevated LH levels, increased GnRH and kisspeptin expression in the hypothalamus, an augmented reproductive organ coefficient, and the early onset of the estrous cycle (26). In our experiment, a similar trend in results was observed, a significantly increased expression of *GnRH* mRNA, *Kiss1* mRNA, and kisspeptin in the model group than in the normal group. The NYRF herbal mixture could effectively delay the advanced puberty by downregulating GnRH and kisspeptin expression, as well as the regulation of LH levels. Surprisingly, no significant differences were observed in E2 levels between groups at PD30. Previous studies have indicated that sex hormones could regulate the expression of *Kiss1* mRNA in different forebrain nuclei, with diametrically opposite trends in the arcuate nucleus and anterior ventricle. High levels of E2 and progesterone can inhibit GnRH release by suppressing *Kiss1* expression in the arcuate nucleus. Interestingly, in the AVPV, elevated levels of E2 stimulate *Kiss1* expression, which releases GnRH (27).

According to this background, in our study, the secretion of E2 levels in rats in 4 groups should be under complex regulation. During the onset of puberty, while E2 levels initially rise gradually, a sharp reduction weakens its inhibitory effect.

Consequently, kisspeptin and GnRH levels increase, leading to elevated E2 secretion. Once E2 reaches its peak, it decreases and fluctuates randomly throughout the estrus cycle (28). Due to the early onset of puberty in rats with PP, they exhibit an earlier peak in E2 levels. In contrast, normal rats are still in the ascending phase before puberty onset. This may explain the lack of significant differences in E2 levels between the model group and the normal group at PD30.

As body weight affects the onset of puberty and vice versa, the effect of the NYRF herbal mixture on body weight was assessed in our study (29). In our study, no difference in body weight was observed in the 4 groups on the day of PD5 and PD30 because the NYRF herbal mixture had no negative effect on body weight, which was consistent with the study by Huang et al. in a rat model of PP (14).

There are some limitations in our study. We only investigated the effects of NYRF on the *RFRP3/GPR147* signal pathway in PP rats induced by danazol. It is still unclear whether these effects would be observed in other animal models or in humans, particularly considering the effects of nutrients and environmental pollution. In future studies, we will apply other different models of PP for in-depth exploration of the deeper mechanisms of the NYRF herbal mixture on *RFRP3/GPR147* signaling pathway.

## 5. Conclusion

Overall, our study highlights the role of the *RFRP3/GPR147* signaling pathway in regulating puberty onset. Upregulation of *RFRP3* may contribute to delaying PP, potentially through the modulation of the hypothalamic *RFRP3/GPR147* signaling pathway. PP rats treated with the NYRF herbal mixture showed lower expression levels of RFRP3. This suggests that the NYRF herbal mixture has the potential to delay the progression of sexual development in rats with PP by the upregulation of *RFRP3* and downregulation of GnRH and kisspeptin in the hypothalamus. These findings provided a deeper understanding of the therapeutic mechanism of the NYRF herbal mixture.

##  Data availability

Research data is not shared.

##  Author contributions

Xiaoli Dai and Yuanyuan He designed the study, conducted the research and wrote the paper. Suhuan Li, Yanyan Sun, and Wen Sun monitored, evaluated, and analyzed the result of the study. Yonghong Wang concepted, designed and reviewed the article. Further, Zhanzhuang Tian, Jian Yu, and Nurgul Yerkinkyzy Ablakimova reviewed the article. All authors approved the final manuscript and take responsibility for the integrity of the data.

##  Conflict of Interest

The authors declare that there is no conflict of interest.
